# ECG-Based Techniques to Optimize Peripherally Inserted Central Catheters: Rationale for Tip Positioning and Practical Use

**DOI:** 10.3389/fcvm.2022.765935

**Published:** 2022-05-06

**Authors:** Giuseppe Gullo, Salah D. Qanadli

**Affiliations:** ^1^Department of Diagnostic and Interventional Radiology, Cardiothoracic and Vascular Unit, Lausanne University Hospital (CHUV), Lausanne, Switzerland; ^2^Faculty of Biology and Medicine (FBM), University of Lausanne (UNIL), Lausanne, Switzerland

**Keywords:** intracavitary electrocardiogram, tip locating device, tip location, PICC, ECG, central venous access, central venous catheters

## Abstract

Intracavitary electrocardiography is an accurate guidance technique for peripherally inserted central catheters (PICC) tip location that is spreading widely among providers using non x-ray-based facilities. The principle behind this technology relies on the transmission of the electrocardiographic signal at the tip of the catheter and its use as an internal mobile electrode, allowing the system to identify the cavo-atrial junction (CAJ) through internal P-wave amplitude modulations. The gain in popularity of intracavitary electrography and its large diffusion have led manufacturers to offer various devices with heterogeneous properties, among which clinician who place PICCs have to choose. It is therefore important to understand differences between available techniques and devices. The potential impact might not only affect availability and costs but also the clinical impact through advantages and limitations regarding electric signal transmission PICC selection. Current perspectives on intracavitary electrocardiography will also be discussed, to give the reader a global view of the management of electrocardiographically guided PICCs, especially in an environment without x-ray support.

## Introduction

The peripherally inserted central catheter (PICC) is a very commonly used medical device. As PICCs are peripherally inserted, the catheter needs to be guided from its insertion point to the central venous system. Currently, this is done using fluoroscopy-guided insertion (FGT), blind technique (BT), or electrocardiogram (ECG) based techniques.

Fluoroscopy-guided insertion uses x-ray fluoroscopy and is considered the gold standard (1–3). By design, this technique needs dedicated infrastructure and technology. The BT is insertion with no guidance and is the most popular, as it can be used bedside with no special technology (4).

More recently, ECG-based techniques that rely on the modification of an intracavitary ECG signal to position the catheter tip optimally in the central vein have been developed (5). The use of these techniques is spreading among non-radiologic users, with a global use of 62% (6).

This manuscript aims to review the rationale for optimal positioning of the catheter tip in the superior vena cava (SVC) and describes the current technologies of ECG-based techniques and their respective advantages and limitations.

In order not to miss a major information, this narrative review integers at least the articles retrieved from Pubmed with the simplified research equation.

Search: (”Catheterization, Peripheral”[Mesh]) AND “Electrocardiography”[Mesh] Filters: English, from 2008–2022.

## Rationale for Optimal Tip Position

Peripherally inserted central catheters has always inspired numerous discussions of complications such as occlusion, infection, and thrombosis (7).

Among these issues, it is known that positioning an internal catheter tip distant from the cavo-atrial junction (CAJ) makes the catheter more likely to undergo internal repositioning and venous thrombosis. As the distance from the catheter tip to the CAJ is the single and strong predictor of central thrombosis and tip repositioning (8), an optimal central tip position is mandatory. This is of particular importance in the pediatric population where blind placements may only reach a 40% of correct positioning (9, 10). Ensuring the central positioning of the catheter has been demonstrated to reduce the risk of PICC-related thrombosis by about 40% (11).

An intracardiac tip position too far from the CAJ may also be a source of unwanted events such as cardiac arrhythmias (tachycardia or bradycardia) due to direct irritation of the right atrium or ventricle (12–14), which is potentially lethal. Catheter placement more than 3–5 cm beyond the CAJ should thus be avoided (12, 15, 16).

**FIGURE 1:**
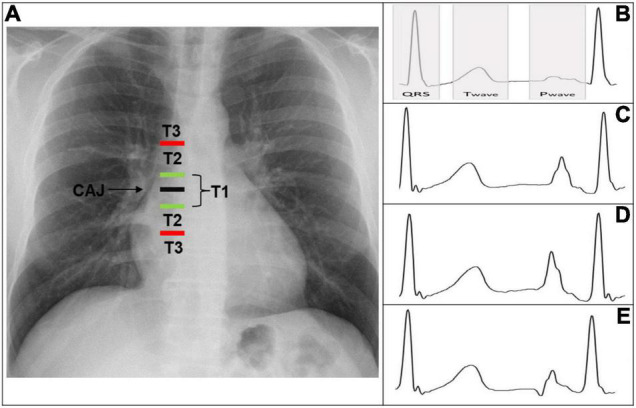
**(A)** Chest X-ray displaying CAJ localization. Catheter tip in T1 (tip within 1 cm of the CAJ) is optimal. Catheter tip in T2 (tip within 1–3 cm of the CAJ) is suboptimal. Catheter tip in T3 (tip more than 3 cm below the CAJ or not in the SVC) is inadequate, needing repositioning. **(B)** Intracavitary electrocardiogram with overlay of the QRS complex zone, T-wave zone, and P-wave zone. A catheter tip in T3 that is not in the SVC will feature this trace, equivalent to the superficial ECG trace. **(C)** Intracavitary electrocardiogram with increasing P-wave (equivalent to the T wave). This is representative of a catheter tip approaching the CAJ in T2. **(D)** Intracavitary electrocardiogram with maximal P-wave without initial negative deflection. The tip is optimally positioned at the CAJ in the T1 zone (within 1 cm of the CAJ). **(E)** Intracavitary electrocardiogram with decreasing P-wave and initial negative deflection. This is representative of a catheter tip beyond the CAJ, in the T2–T3 zone. CAJ, cavo-atrial junction; SVC, superior vena cava.

According to the distance from the tip to the CAJ, the PICC positions can be classified into three types (Figure 1A) (4).

1. T1: Tip is within 1 cm of the CAJ (optimal).
2. T2: Tip is between 1 and 3 cm from the CAJ (suboptimal).
3. T3: Tip is >3 cm below the CAJ or is not in the SVC (inadequate, needing repositioning).

## Principle of ECG-Based Techniques

The electrocardiogram localization technology relies on the use of Lead II’s complex of Einthoven’s Triangle in order to obtain the maximal P-wave signal [limb electrodes at the right arm (RA) and left leg (LL)] (17).

This surface tracing serves as reference and is compared to the same tracing seen in intracavitary through the PICC tip, which is used as an electrode.

The P-wave varies between the two tracing reaching its highest value at the CAJ.

The operator will therefore be able to find the position of the catheter related to the maximum height of the P-wave.

## Differentiating Catheters

Peripherally inserted central catheters are not a uniform product, there are a lot of subtle and less subtle variations on PICC design that deserve to be addressed.

The actual catheters can be differentiated based on their material which is mostly polyurethane (PUR) but also silicone.

They may also be differentiated by the location of the cut to adjust their length. Some catheters are trimmed on their “free end side” which is the proximal part of the catheter, closer to the hearth. While other are trimmed on their outer part, near to the hub, which is the distal part of the catheter.

The distal catheter part may also vary regarding its design. It may be straight, i.e., with the same French size on the whole catheter or it may be “reverse tapered” which consists in a gradual augmentation of the French catheter size in the last centimeters of the catheter (generally 2 more French size in 5–8 centimeters length).

Some catheters are non-valved while others are valved this permits aspiration with negative pressure, infusion with positive pressure and a closed system with neutral pressure.

Among these, the strongest point of attention is the trim point which further determines the technique of insertion.

## Technical Aspects

### Insertion Techniques

There are two procedural techniques varying depending on the trim point (proximal or distal).

#### Device With Proximal Trim Point (Technique A)

The patient is prepared using the standard maximal sterile barrier approach with external ECG electrodes placed the chest.

The catheter is inserted into one of the upper arm veins using an ultrasound-guided out-of-plane control and modified Seldinger technique (insertion of a small-gauge needle into the vein followed by a wire; sheath dilators are used for the catheter to gain access to the vein), with the arm abducted to approximately 75–90°.

Before insertion, the catheter is trimmed to the approximate anticipated required length. This approximation is based on the following calculation, using anthropometric measurements: insertion site to axillary crease distance + axillary crease to sternal notch distance + sternal notch to the third intercostal space (4). The free-end side (inner side) of the PICC is cut using a scalpel or scissors.

The PICC is advanced into the central circulation. On the display interface, both surface (i.e., ECG waveforms received from the patient’s skin) and intracavitary (ECG signals from the tip of the catheter) ECG traces are visible in real time, allowing the operator to see if the PICC is taking the correct path toward the heart. This is done by following P-wave modulations (see intracavitary ECG paragraph). The conductivity is ensured through either a solid or liquid medium (see conductivity paragraph).

The tip is implanted at the CAJ and the puncture site is dressed using a catheter stabilization device (18).

**FIGURE 2:**
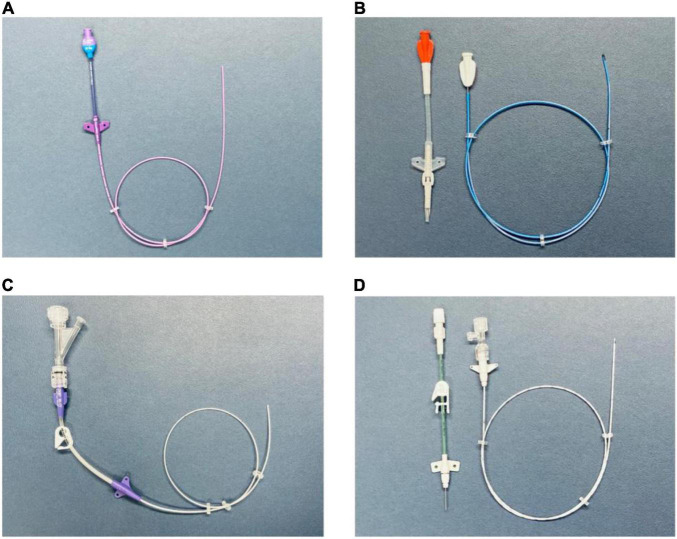
**(A)** PICC with a free-end-side trim point (technique A). Valved reverse-tapered device with solid-wire signal conduction. PUR material with high-flow injection properties. **(B)** PICC with a hub-side (outer part) trim point (technique B). Centrally valved straight device with solid-wire signal conduction and silicone material without high-flow injection properties. **(C)** PICC with a free-end-side trim point (technique A). Non-valved reverse-tapered device with liquid (saline) signal conduction. PUR material with high-flow injection properties. **(D)** PICC with a hub-side (outer part) trim point (technique B). Non-valved straight device with liquid (saline) signal conduction. PUR material with high-flow injection properties. PICC, peripherally inserted central catheter; PUR, polyurethane.

This technique allows the manufacturer to design the device with features such as a reverse-tapered hub-side catheter with a fixed, integrated valve (19, 20) (Figures 2A,C).

#### Device With Distal (Outer Part) Trim Point (Technique B)

The patient preparation, venous access, ECG following and PICC advancing into the central circulation, and site dressing are identical to those in technique A.

The PICC is not trimmed before to advance it into the central circulation but the approximate anticipated required length is used to identify the position where a P-wave modification should happen.

After the PICC implantation at the CAJ following P-wave modulations, the distal part of the PICC is then cut without the need to retire it and the removable part of the catheter is then connected.

This technique allows the manufacturer to use tapered, open-ended distal tips or valved, closed distal tips (21) (Figures 2B,D).

### Tip Positioning

Thera are two distinct phase in tip positioning (22), the navigation phase that consists in directing the PICC from the insertion point to the SVC and the locating phase consisting in accurately setting the tip at the CAJ.

#### Navigation

Tip navigation (using a reference electrode) consists in verifying the correct direction of the catheter. This can be achieved through the interpretations of the components of QRS waves allowing differentiation of non-central veins (brachial/basilic/innominate/subclavian/internal-jugular). Thus it is rather complicated (23).

Magnetic tip navigation is linked to a unique device (Sherlock-3CG^®^) and instead of using the QRS complex, it uses the catheter tip and the Y shield as a low field magnetic complex. The position of the tip relative to the shield is electronically displayed on a screen, allowing the operator to follow the tip moves in real time toward SVC and identifying the wrong locations such as neck or contralateral side (24).

The two techniques are very different, with the magnetic technology being much more intuitive than the QRS due to the real time following of the tip.

These technologies haven’t been directly compared. The only data available present a success rate of 80%, 10% of unstable/unreadable cases and 10% of wrong information for magnetic technology (22).

#### Intracavitary ECG

The ECG monitor displays an indirect view of the catheter’s position. This is an evolutive real-time sequence that relies on the modification of the intracavitary P-wave shape and amplitude relative to the surface ECG tracing.

(a) As long as the catheter is distant from the central position, the P-wave remains unchanged and is equivalent to the superficial ECG trace.

The tip is not in the SVC, corresponding to a T3 position (Figure 1B).

(b) As the tip nears the CAJ, the intracavitary P-wave slowly increases in amplitude.

The tip is within 3 cm of the CAJ, corresponding to a T2 position (Figure 1C).

(c) At the moment the catheter is at the CAJ level, the P-wave will show its maximal amplitude without initial negative deflection (Figure 1D).

The tip is within 1 cm of the CAJ, corresponding to a T1 position (Figure 1D).

(d) When the tip proceeds further than the CAJ, the P-wave will shrink again and present an initial negative deflection (Figure 1E) (25). The tip is far beyond the CAJ, corresponding to a T2–T3 position (Figure 1E).

When the amplitude of the P-wave is more than 50% of the R part from QRS complex, the accuracy rate of PICC placement is superior to 99% (26).

In order to integrates the catheter moves between abduction and adduction Zhu et al. (27) suggest to place the catheter with the peak P-wave with the arm in adduction.

### ECG Signal Transmission (Conductivity)

The signal transmission between the catheter tip and lead may be achieved by using either a solid (Figures 2A,B) or liquid medium (Figures 2C,D).

The solid medium is a metallic guide wire, while the liquid medium is generally saline water (28). Sodium bicarbonate solution (29) as also been mentioned in the literature but it doesn’t seem to be actually used.

The coupling of the ECG monitor to the PICC may be achieved through commercially available wire-based (30) or saline solution–based devices (31), or through homemade devices (connectors and a standard ECG machine) (32–34).

Several studies have compared ECG technologies with regard to their intracavitary electrode type (liquid vs. wire) and their capacity to identify the CAJ. The results from multiple implantable devices have been synthesized in a meta-analysis (35) that showed an absence of statistically significant difference in tip location placement.

The way conductivity is achieved may also have some importance in the PICC placement. When comparing wire vs. saline, some (36) have advanced the view that wire technology creates errors of precision due to loss of catheter rigidity and subsequent change in its course. This was evidenced in left-sided access as the route is longer and more tortuous. Thus, the authors recommended advancing the catheter an additional 1–2 cm. Monard et al. also mentioned problems with the left sided access (37).

Due to its importance, the central positioning of the tip should be the primary goal of each intervention.

### Material

The longer the catheter stays implanted, the more it is susceptible to barium sulfate (BaSO_4_) particle loss, which creates weak points and makes silicone more prone to mechanical failures (38). As most of the patients are likely to have computed tomography exams, the clear trend is toward the use of PUR lines, as they support 5 mL/s high pressure injections (39) while silicone can at the best case support 3 mL/s. Silicone devices are also more expensive.

## Risk of Complications

At insertion point the reverse tapered catheter is designed to give kinking resistance as well as participate to a better hemostasis which is a rather interesting property.

However, Itkin et al. (19), in a randomized study addressing straight and tapered devices, mentioned no statistical difference regarding safety endpoints such as post-procedure bleeding rates and thrombosis incidence. No other comparison point exists, as theirs is the only such study.

Technique A presents the interesting possibility of integrating valves in the hub side, making clamping unnecessary. The valve is bidirectional, opening inwardly during infusion and outwardly during aspiration; this minimizes occlusion rates due to blood reflux in the catheter and subsequent clotting, by 20% (20, 40). Two more recent RCT tried to address the effect of valve technology on occlusion rate with no advantages of valved vs. non-valved PICC, however both have interrupted for other reasons (21, 41).

Also, cutting the open end (technique A) results in the loss of some of the manufactured smooth and round catheter tip (42). This introduction of “roughness” is of no importance in technique B but may have some clinical repercussions, such as an increased risk of thrombosis or infection, with technique A (43). However no studies have addressed the problem of PICC tip roughness and thrombosis or infections.

Intuitively, it makes sense that a rough catheter tip may not be ideal. Another option exists, combining technique B and a distally (inner) valved closed tip. This catheter configuration seems similar to an outer valve in regard to occlusion rates (44). The only device with a distal valve is made out of silicone (which is probably the only way to have an inner valve) whereas most of the other actual devices are made of PUR.

The complication rates of silicone and PUR devices are apparently similar, though it is difficult to thoroughly address this point due to a lacunar specification of post-care informations in studies (39).

## Cost Effectiveness

It exists no points of comparison between ECG devices, in that way ECG technologies will be discussed relative to other techniques.

### Productivity

Interventional radiology services are usually overwhelmed by PICC demands, delaying patient care and lowering discharge rates for outpatient therapies. This is usually linked to the fact that a PICC is regarded as a “last-in-line procedure” with no degree of priority vs. life-saving or complex interventional procedures (45, 46).

The ECG system is very powerful in its capacity to improve productivity. The radiology outsourcing may lower organizational costs such as waiting delay for insertion from 6 times (46) and ECG may eliminate the time needed to confirm correct device placement through X-ray (47).

### Costs

When facing structural costs, the use of ECG technology is a straightforward means of budget containment (48, 49), with a decrease in expenses of €30000–€50000 (US $35000–$58000) per year, relative to an operating room (50). Relative to an interventional radiology suite, the decrease in expenses may be in the range of US $150–200 per hour (51). It is also cost effective when compared to blind placements (assuming the replacement rates) with a cost of US $ 318 per procedure vs. US $ 384 (52).

### Skill Substitution

In regard to medical skill substitution, ECG technology may be a good alternative when professionals have limited access to fluoroscopic devices, it may also provide a way to accommodate restrictions on the use of fluoroscopy by non-licensed practitioners such as PICC nurses (53). Skill substitution also favors the creation of specifically dedicated vascular access teams (VAT). PICC management realized by the VAT may be considered an advanced practice allowing lower complications rate and raising overall satisfaction. This can also allow faster intervention time as well as lower costs, especially linked to physician substitution (54).

## Discussion

Considering liquid transmission, saline has half the conductivity of sodium bicarbonate (NaHCO_3_), (respective impedances are 200,000 and 100,000 Ω). When it is needed to enhance the ECG signal capacity, an hypertonic solution may be effective, Capasso et al. (55) used a 4% saline which has an impedance of is 77,000 Ω, similar to that of NaHCO_3_ (56). Regarding wire transmission, high conductivity is characteristic of metals. Enhancing saline concentration enhances the conductivity but care must be taken in the ability of kidneys to excrete sodium (in newborns this capacity is low) (57).

Despite this, the analysis was markedly lacking in evidence because the retained studies were only quasi-experimental in design. Moreover, the included studies concern mostly central venous catheters (CVC) such as port, jugular CVC, or tunneled CVC. More powerful trials oriented to PICCs should be run.

Stenosis and obstructions are rather uncommon (3%) in populations without catheter history but may rise to 7.5% in populations with central catheterization history (58). In these cases, mapping the SVC system through venography is a necessary step for a successful guidance procedure as well as for endoluminal interventions such as balloon angioplasty. As well, resistance during the passage of the catheter with extravascular wire tracking indicates venous perforation risk (59) and also necessitates cartography through venography (60).

These elements may clearly be considered a limiting factor of ECG technologies. Fluoroscopy permits a direct tract visualization which is greatly useful in identifying the right venous pathway which may be challenging to identify through indirect ECG visualization (61). Arterial pathway may also not be highlighted through indirect ECG guidance (62).

At the same time, the scope of use of ECG guidance is constantly increasing in connection with clinical research and former limits of the system may be alleviated. Even if active magnetic device such as ventricular assist device are still problematic for ECG guidance (63), concomitant indwelling central catheters has been proved not to affect tip positioning (64). Also it has been showed that patients presenting abnormal surface ECG such as atrial fibrillation may be successfully managed through the use of ECG guidance (65). This technique may also be supplemented by the “bubble test.” In correct placed PICCs, it consists in ultrasound visualization of micro-bubbles in the right atrium (subcostal four chamber view) within 2 s after injection of saline–air mixture (66).

Considering secondary malposition diagnosis (tip migration after an initial adequate central positioning). Saline ECCG technology allows the following of the inserted tip in a way as effective as diagnostic CRX (67) and bedside repositioning through P-Wave morphology control after each repositioning maneuvers (arm abduction, high flow flush technique, patient mobilization) (68).

What precision can be reached under ECG is a frequent subject of controversy between users who are more for the use of FX technology or more for the use of ECG technology (2, 3, 28). What is undeniable is that ECG is clearly superior to blind insertion techniques (69, 70).

On one hand, ECG is especially interesting for premature infants as accumulated radiation dose may manifest years after exposure (71). On the other hand, in order to reach the same degree of central tip precision under ECG as under fluoroscopy, accommodations regarding the insertion point should be acknowledged.

The first kind of concession (technique A) is related to the length of catheter needed to ensure the capacity to observe the P-wave modifications (P-wave maximal amplitude in the absence of initial deflection) and by this means to position the catheter at the CAJ. Operators may need up to 2 cm of extra catheter length (72) while Elli put forward a 3.8 cm discrepancy between ECG measure and cutaneous landmark (73); ultimately, these leeway centimeters will reverberate at the insertion point (30) and may have uncertain repercussions (74).

The second type of concession is related to the disturbances caused by the device at the insertion point. These disturbances are either intravascular or extravascular.

Should the device be reverse tapered to ensure better hemostasis (technique A), its increase in diameter (toward the hub) may be as high as 2F, with potential associated bloodstream perturbations (75).

The different advantages and limits of PICC devices classified by technique and transmission medium are summarized in Table 1. Table 2 summarizes different commercially available catheters classified by technique and valve presence.

**TABLE 1:** Summary of the different advantages and limits of PICC devices classified by technique and transmission medium.

	Technique A		Technique B	
Wire	Precision	✓	Precision	✓
	Clot occlusion of the PICC	✓	Clot occlusion of the PICC	✓
	Conduction	✓✓	Conduction	✓✓
	Insertion site repercussions	–	Insertion site repercussions	✓
	5 ml/s power injection	✓	5 ml/s power injection	–
	Cost	✓	Cost	–
Saline	Precision	✓	Precision	✓
	Clot occlusion of the PICC	–	Clot occlusion of the PICC	–
	Conduction	✓	Conduction	✓
	Insertion site repercussions	–	Insertion site repercussions	✓
	5 ml/s power injection	✓	5 ml/s power injection	✓
	Cost	✓	Cost	✓

**TABLE 2:** Summary of different commercially available PICC devices classified by technique and valve presence.

	Technique A	Technique B
Valved	• Valved Pro-PICC^®^; Medical Components, Inc., Harleysville, PA, United States • BioFlo™ PICC with Endexo and PASV Valve Technology; AngioDynamics Latham, NY, United States • PowerPICC SOLO™2; Becton Dickinson, Franklin Lakes, NJ, United States*	• PowerGroshong™ PICC Catheter; Becton Dickinson, Franklin Lakes, NJ, United States*
Non-valved	• Synergy CT PICC™; Health Line Medical Products, Salt Lake City, UT, United States • Turbo-Ject^®^ Power-Injectable; Cook Medical, Bloomington, IN, United States • Arrowg + ard Blue Advance™ PICC; Teleflex Incorporated, Wayne, PA, United States* • Celsite^®^ PICC-Cel; B. Braun Melsungen AG, Hessen, Germany	• CT PICC^®^ Easy; Vygon, Ecouen, France**

All device may be ECG guided through homemade positioning system using connectors and a standard ECG machine with saline or wire transmission medium.

*Device with brand catheter tip positioning system, wired transmission medium.

**Device with brand catheter tip positioning system, saline transmission medium.

## Conclusion

In practical use, a PICC and ECG can be combined in multiple ways, depending on the device’s trim point, material, valve position, and transmission medium, according to the operator’s choices. From a strategic point of view, the ECG-guided PICC is an effective alternative oriented toward undelayed patient management and optimized workflow, although it lacks some strengths that are needed in complex situations.

## Author Contributions

SQ: conceptualization, critical review, editing of the draft, and decision to submit for publication. GG: preparation, visualization, data presentation, writing the initial draft, editing of the draft, and creation of the published work. Both authors contributed to the article and approved the submitted version.

## Conflict of Interest

The authors declare that the research was conducted in the absence of any commercial or financial relationships that could be construed as a potential conflict of interest.

## Publisher’s Note

All claims expressed in this article are solely those of the authors and do not necessarily represent those of their affiliated organizations, or those of the publisher, the editors and the reviewers. Any product that may be evaluated in this article, or claim that may be made by its manufacturer, is not guaranteed or endorsed by the publisher.

## References

[1] Chrisman HB Omary RA Nemcek AA Ryu RK Saker MB Vogelzang RL. Peripherally inserted central catheters: guidance with use of US versus venography in 2,650 patients. *J Vasc Interv Radiol JVIR.* (1999) 10:473–5. 10.1016/s1051-0443(99)70067-9 10229477

[2] Gullo G Colin A Frossard P Jouannic AM Knebel JF Qanadli SD. Appropriateness of replacing fluoroscopic guidance with ECG-electromagnetic guidance for PICC insertion: a randomized controlled trial. *AJR Am J Roentgenol.* (2021) 216:981–8. 10.2214/AJR.20.23345 33594912

[3] Mack V Nißler D Kasikci D Malouhi A Aschenbach R Teichgräber U. Magnetic tracking and electrocardiography-guided tip confirmation system versus fluoroscopy for placement of peripherally inserted central catheters: a randomized, noninferiority comparison. *Cardiovasc Intervent Radiol.* (2020) 43:1891–7. 10.1007/s00270-020-02551-0 32556606 PMC7649160

[4] Glauser F Breault S Rigamonti F Sotiriadis C Jouannic A-M Qanadli SD. Tip malposition of peripherally inserted central catheters: a prospective randomized controlled trial to compare bedside insertion to fluoroscopically guided placement. *Eur Radiol.* (2017) 27:2843–9. 10.1007/s00330-016-4666-y 27957644

[5] Pittiruti M Scoppettuolo G La Greca A Emoli A Brutti A Migliorini I The EKG method for positioning the tip of PICCs: results from two preliminary studies. *J Assoc Vasc Access.* (2008) 13:179–86. 10.2309/java.13-4-4

[6] Chopra V Kuhn L Ratz D Winter S Carr PJ Paje D Variation in use of technology among vascular access specialists: an analysis of the PICC1 survey. *J Vasc Access.* (2017) 18:243–9. 10.5301/jva.5000711 28430309

[7] Vesely TM. Central venous catheter tip position: a continuing controversy. *J Vasc Interv Radiol JVIR.* (2003) 14:527–34. 10.1097/01.rvi.0000071097.76348.72 12761305

[8] Ballard DH Samra NS Gifford KM Roller R Wolfe BM Owings JT. Distance of the internal central venous catheter tip from the right atrium is positively correlated with central venous thrombosis. *Emerg Radiol.* (2016) 23:269–73. 10.1007/s10140-016-1393-2 27112774

[9] Mundi MS Varayil JE McMahon MT Okano A Vallumsetla N Bonnes SL Accuracy of intravenous electrocardiography confirmation of peripherally inserted central catheter for parenteral nutrition. *Nutr Clin Pract.* (2016) 31:207–10. 10.1177/0884533615621548 26850037

[10] Zhou L Xu H Liang J Xu M Yu J. Effectiveness of intracavitary electrocardiogram guidance in peripherally inserted central catheter tip placement in neonates. *J Perinat Neonatal Nurs.* (2017) 31:326–31. 10.1097/jpn.0000000000000264 28520655

[11] Meyer BM. Developing an alternative workflow model for peripherally inserted central catheter placement. *J Infus Nurs.* (2012) 35:34–42. 10.1097/NAN.0b013e31823bc8fd 22222290

[12] Hacking MB Brown J Chisholm DG. Position dependent ventricular tachycardia in two children with peripherally inserted central catheters (PICCs). *Pediatr Anesth.* (2003) 13:527–9. 10.1046/j.1460-9592.2003.01021.x 12846710

[13] Nazinitsky A Covington M Littmann L. Sinus arrest and asystole caused by a peripherally inserted central catheter. *Ann Noninvasive Electrocardiol.* (2013) 19:391–4. 10.1111/anec.12116 24286255 PMC6932029

[14] Thyoka M Haq I Hosie G. Supraventricular tachycardia precipitated by a peripherally inserted central catheter in an infant with gastroschisis. *Case Rep.* (2014) 2014:bcr2013201203. 10.1136/bcr-2013-201203 24569259 PMC3939389

[15] Alvarez P Schurmann P Smith M Valderrábano M Lin CH. Position-dependent ventricular tachycardia related to peripherally inserted central venous catheter. *Methodist DeBakey Cardiovasc J.* (2016) 12:177–8. 10.14797/mdcj-12-3-177 27826374 PMC5098577

[16] Bivins MH Callahan MJ. Position-dependent ventricular tachycardia related to a peripherally inserted central catheter. *Mayo Clin Proc.* (2000) 75:414–6. 10.4065/75.4.414 10761499

[17] Chapelon-Abric C. Méthode d’analyse des électrocardiogrammes de surface douze dérivations. *EMC Cardiol Angéiologie.* (2004) 1:97–105. 10.1016/j.emcaa.2004.03.003

[18] Dale M Higgins A Carolan-Rees G. Sherlock 3CG^§^ tip confirmation system for placement of peripherally inserted central catheters: a NICE medical technology guidance. *Appl Health Econ Health Policy.* (2015) 14:41–9. 10.1007/s40258-015-0192-3 26293389 PMC4740556

[19] Itkin M Mondshein JI Stavropoulos SW Shlansky-Goldberg RD Soulen MC Trerotola SO. Peripherally inserted central catheter thrombosis—reverse tapered versus nontapered catheters: a randomized controlled study. *J Vasc Interv Radiol.* (2014) 25:85–91.e1. 10.1016/j.jvir.2013.10.009 24268631

[20] Hoffer EK Borsa J Santulli P Bloch R Fontaine AB. Prospective randomized comparison of valved versus nonvalved peripherally inserted central vein catheters. *Am J Roentgenol.* (1999) 173:1393–8. 10.2214/ajr.173.5.10541127 10541127

[21] Johnston AJ Streater CT Noorani R Crofts JL Del Mundo AB Parker RA. The effect of peripherally inserted central catheter (PICC) valve technology on catheter occlusion rates – the ‘ELeCTRiC’ study. *J Vasc Access.* (2012) 13:421–5. 10.5301/jva.5000071 22505280

[22] Pittiruti M Scoppettuolo G Dolcetti L Emoli A. Clinical use of Sherlock-3CG^®^ for positioning peripherally inserted central catheters. *J Vasc Access.* (2019) 20:356–61. 10.1177/0300060518793802 30334475

[23] Ling Q Chen H Tang M Qu Y Tang B. Accuracy and safety study of intracavitary electrocardiographic guidance for peripherally inserted central catheter placement in neonates. *J Perinat Neonatal Nurs.* (2019) 33:89–95. 10.1097/JPN.0000000000000389 30676468

[24] Rosche N Stehr W. Evaluation of a magnetic tracking and electrocardiogram-based tip confirmation system for peripherally inserted central catheters in pediatric patients. *J Infus Nurs.* (2018) 41:301–8. 10.1097/NAN.0000000000000293 30188452

[25] Yuan L Li R Meng A Feng Y Wu X Yang Y Superior success rate of intracavitary electrocardiogram guidance for peripherally inserted central catheter placement in patients with cancer: a randomized open-label controlled multicenter study. *PLoS One.* (2017) 12:e0171630. 10.1371/journal.pone.0171630 28278167 PMC5344315

[26] Dong H-M Zhu Y-X Yin X-X Zhang X. Clinical significance of different atlas of intracavitary electrocardiogram for PICC localization in 961 cases. *Ann Noninvasive Electrocardiol.* (2021) 27:e12904. 10.1111/anec.12904 34825734 PMC8739621

[27] Zhu S-S Zhao J Zhou X-Y Gao W Pan M-H Yu W-W Influence of arm position change from adduction to abduction on intracavitary electrocardiogram. *J Vasc Access.* (2021) 22:292–8. 10.1177/1129729819891565 31808719

[28] Moureau NL Dennis GL Ames E Severe R. Electrocardiogram (EKG) guided peripherally inserted central catheter placement and tip position: results of a trial to replace radiological confirmation. *J Assoc Vasc Access.* (2010) 15:8–14. 10.2309/java.15-1-3

[29] Cheng KI Chu KS Chen LT Tang CS. Correct positioning of the venous port-a-cath catheter: comparison of intravascular electrocardiography signal from guidewire and sodium bicarbonate flushed catheter. *Anaesth Intensive Care.* (2002) 30:603–7. 10.1177/0310057X0203000510 12413260

[30] Johnston AJ Holder A Bishop SM See TC Streater CT. Evaluation of the sherlock 3CG tip confirmation system on peripherally inserted central catheter malposition rates. *Anaesthesia.* (2014) 69:1322–30. 10.1111/anae.12785 25040430

[31] Pittiruti M Bertollo D Briglia E Buononato M Capozzoli G De Simone L The intracavitary ECG method for positioning the tip of central venous catheters: results of an Italian multicenter study. *J Vasc Access.* (2012) 13:357–65. 10.5301/JVA.2012.9020 22328361

[32] Baldinelli F Capozzoli G Pedrazzoli R Marzano N. Evaluation of the correct position of peripherally inserted central catheters: anatomical landmark vs. electrocardiographic technique. *J Vasc Access.* (2015) 16:394–8. 10.5301/jva.5000431 26109544

[33] Cales YK Rheingans J Steves J Moretti M. Electrocardiogram-guided peripherally inserted central catheter tip confirmation using a standard electrocardiogram machine and a wide-mouth electrocardiogram clip compared with traditional chest radiograph. *J Assoc Vasc Access.* (2016) 21:44–54. 10.1016/j.java.2015.12.001

[34] Zhao R Chen C Jin J Sharma K Jiang N Shentu Y Clinical evaluation of the use of an intracardiac electrocardiogram to guide the tip positioning of peripherally inserted central catheters. *Int J Nurs Pract.* (2016) 22:217–23. 10.1111/ijn.12409 26617329

[35] Ling G Zhiwen W Guorong W Shaomei S Xue W. Guide wire electrode versus liquid electrode for intravascular electrocardiography-guided central venous catheterization in adults: a systematic review and meta-analysis. *J Vasc Access.* (2020) 21:564–72. 10.1177/1129729819868044 31422729

[36] Kremser J Kleemann F Reinhart K Schummer W. Optimized method for correct left-sided central venous catheter placement under electrocardiographic guidance. *Br J Anaesth.* (2011) 107:567–72. 10.1093/bja/aer189 21697183

[37] Monard C Lefèvre M Subtil F Piriou V David J-S. Peripherally inserted central catheter with intracavitary electrocardiogram guidance: malposition risk factors and indications for post-procedural control. *J Vasc Access.* (2018) 20:128–33. 10.1177/1129729818781266 29936885

[38] Braun U Lorenz E Weimann C Sturm H Karimov I Ettl J Mechanic and surface properties of central-venous port catheters after removal: a comparison of polyurethane and silicon rubber materials. *J Mech Behav Biomed Mater.* (2016) 64:281–91. 10.1016/j.jmbbm.2016.08.002 27552159

[39] Seckold T Walker S Dwyer T. A comparison of silicone and polyurethane PICC lines and postinsertion complication rates: a systematic review. *J Vasc Access.* (2015) 16:167–77. 10.5301/jva.5000330 25634150

[40] Hinson EK Blough LD. Skilled IV therapy clinicians’ product evaluation of open-ended versus closed-ended valve PICC lines: a cost savings clinical report. *J Intraven Nurs.* (1996) 19:198–210. 8852178

[41] Pittiruti M Emoli A Porta P Marche B DeAngelis R Scoppettuolo G. A prospective, randomized comparison of three different types of valved and non-valved peripherally inserted central catheters. *J Vasc Access.* (2014) 15:519–23. 10.5301/jva.5000280 25198813

[42] Jegatheeswaran A Parmar N Walton JM Yip C Chan AKC. Quantitative analysis of catheter roughness induced by cutting and manipulation: a potential prothrombotic risk. *Blood Coagul Fibrinolysis.* (2007) 18:531–6. 10.1097/MBC.0b013e3282010ae6 17762527

[43] Parvez B Parmar N Chan AKC. Trimming of peripherally inserted central venous catheters may increase the risk of thrombosis. *Thromb Res.* (2004) 113:175–7. 10.1016/j.thromres.2004.02.013 15115673

[44] Ong CK Venkatesh SK Lau GB Wang SC. Prospective randomized comparative evaluation of proximal valve polyurethane and distal valve silicone peripherally inserted central catheters. *J Vasc Interv Radiol JVIR.* (2010) 21:1191–6. 10.1016/j.jvir.2010.04.020 20598573

[45] Chan-Dinevski I Annamalai G. Peripherally inserted central catheters (PICCs) at the bedside by X-ray technologists: a review of our experience. *J Med Imaging Radiat Sci.* (2020) 51:373–8. 10.1016/j.jmir.2020.05.003 32800675

[46] Robinson MK Mogensen KM Grudinskas GF Kohler S Jacobs DO. Improved care and reduced costs for patients requiring peripherally inserted central catheters: the role of bedside ultrasound and a dedicated team. *JPEN J Parenter Enteral Nutr.* (2005) 29:374–9. 10.1177/0148607105029005374 16107601

[47] Walker G Todd A. Nurse-led PICC insertion: is it cost effective? *Br J Nurs Mark Allen Publ.* (2013) 22:S9–15. 10.12968/bjon.2013.22.Sup19.S9 24350393

[48] Oliver G Jones M. Evaluation of an electrocardiograph-based PICC tip verification system. *Br J Nurs Mark Allen Publ.* (2013) 22:S24–8. 10.12968/bjon.2013.22.Sup9.S24 24261004

[49] Oliver G Jones M. ECG-based PICC tip verification system: an evaluation 5 years on. *Br J Nurs.* (2016) 25:S4–10. 10.12968/bjon.2016.25.19.S4 27792447

[50] Bloemen A Daniels AM Samyn MG Janssen RJ Elshof J-W. Electrocardiographic-guided tip positioning technique for peripherally inserted central catheters in a Dutch teaching hospital: feasibility and cost-effectiveness analysis in a prospective cohort study. *J Vasc Access.* (2018) 19:578–84. 10.1177/1129729818764051 29560814

[51] Neuman ML Murphy BD Rosen MP. Bedside placement of peripherally inserted central catheters: a cost-effectiveness analysis. *Radiology.* (1998) 206:423–8. 10.1148/radiology.206.2.9457195 9457195

[52] Keller EJ Aragona E Molina H Lee J Salem R Resnick SA Cost-effectiveness of a guided peripherally inserted central catheter placement system: a single-center cohort study. *J Vasc Interv Radiol JVIR.* (2019) 30:709–14. 10.1016/j.jvir.2018.07.032 30773436

[53] Royce B. Use of C-arm fluoroscopy by nurses for placement of picc lines. *J Assoc Vasc Access.* (2009) 14:138–41. 10.2309/java.14-3-5

[54] Corcuera Martínez MI Aldonza Torres M Díez Revilla AM Maali Centeno S Mañeru Oria A Elizari Roncal I Impact assessment following implementation of a vascular access team. *J Vasc Access.* (2020) 23:135–44. 10.1177/1129729820984284 33356810

[55] Capasso A Mastroianni R Passariello A Palma M Messina F Ansalone A The intracavitary electrocardiography method for positioning the tip of epicutaneous cava catheter in neonates: pilot study. *J Vasc Access.* (2018) 19:542–7. 10.1177/1129729818761292 29552936

[56] Colley PS Artru AA. ECG-guided placement of sorenson CVP catheters via arm veins. *Anesth Analg.* (1984) 63:953–6. 6486495

[57] Yang L Bing X Song L Na C Minghong D Annuo L. Intracavitary electrocardiogram guidance for placement of peripherally inserted central catheters in premature infants. *Medicine (Baltimore).* (2019) 98:e18368. 10.1097/MD.0000000000018368 31852143 PMC6922499

[58] Gonsalves CF Eschelman DJ Sullivan KL DuBois N Bonn J. Incidence of central vein stenosis and occlusion following upper extremity PICC and port placement. *Cardiovasc Intervent Radiol.* (2003) 26:123–7. 10.1007/s00270-002-2628-z 12616419

[59] Bhutta ST Culp WC. Evaluation and management of central venous access complications. *Tech Vasc Interv Radiol.* (2011) 14:217–24. 10.1053/j.tvir.2011.05.003 22099014

[60] Park KB Choo SW Do YS Shin SW Cho SK Choe YH Peripherally inserted central catheter placement in patients with unsuspected central venous obstruction. *J Vasc Interv Radiol.* (2008) 19:552–6. 10.1016/j.jvir.2007.10.010 18375300

[61] Konstantinou EA Sapsakos TDM Katsoulas TA Velecheris D Tsitsimelis D Bonatsos G. Persistent left superior vena cava leads to catheter malposition during PICC port placement. *J Vasc Access.* (2016) 17:e29–31. 10.5301/jva.5000498 26797899

[62] DiLoreto E Bahl A. Inadvertent arterial peripherally inserted central catheter insertion with corresponding electrocardiographic tracings: a case report. *J Infus Nurs.* (2021) 45:37–40. 10.1097/NAN.0000000000000452 34775430

[63] Takakura M Fujii T Suzuki S Nishiwaki K. Interference of a ventricular assist device with magnetic navigation during insertion of sherlock 3CG™, a bedside peripherally inserted central catheter. *J Artif Organs.* (2021). 10.1007/s10047-021-01293-1 34524593

[64] Sun W Li J Liu B Liu Y Ge R Wang K Effects of indwelling centrally inserted central catheter on tip location of peripherally inserted central catheter with intracavitary electrocardiogram guidance: a retrospective case-control study. *J Vasc Access.* (2021). 10.1177/11297298211015088 34296629

[65] Gao Y Liu Y Zhang H Fang F Song L. The safety and accuracy of ECG-guided PICC tip position verification applied in patients with atrial fibrillation. *Ther Clin Risk Manag.* (2018) 14:1075–81. 10.2147/TCRM.S156468 29922068 PMC5995413

[66] Iacobone E Elisei D Gattari D Carbone L Capozzoli G. Transthoracic echocardiography as bedside technique to verify tip location of central venous catheters in patients with atrial arrhythmia. *J Vasc Access.* (2020) 21:861–7. 10.1177/1129729820905200 32126882

[67] Yu T Wu L Yuan L Dawson R Li R Qiu Z The diagnostic value of intracavitary electrocardiogram for verifying tip position of peripherally inserted central catheters in cancer patients: a retrospective multicenter study. *J Vasc Access.* (2019) 20:636–45. 10.1177/1129729819838136 30919741

[68] Weber MD Himebauch AS Conlon T. Repositioning of malpositioned peripherally inserted central catheter lines with the use of intracavitary electrocardiogram: a pediatric case series. *J Vasc Access.* (2020) 21:259–64. 10.1177/1129729819865812 31364466

[69] Liu G Hou W Zhou C Yin Y Lu S Duan C Meta-analysis of intracavitary electrocardiogram guidance for peripherally inserted central catheter placement. *J Vasc Access.* (2019) 20:577–82. 10.1177/1129729819826028 30838913

[70] Yin Y-X Gao W Li X-Y Lu W Deng Q-H Zhao C-Y Randomized multicenter study on long-term complications of peripherally inserted central catheters positioned by electrocardiographic technique. *Phlebology.* (2020) 35:614–22. 10.1177/0268355520921357 32375605

[71] Xiao A Sun J Zhu L Liao Z Shen P Zhao L Effectiveness of intracavitary electrocardiogram-guided peripherally inserted central catheter tip placement in premature infants: a multicentre pre-post intervention study. *Eur J Pediatr.* (2020) 179:439–46. 10.1007/s00431-019-03524-3 31788740

[72] Li W Xu R Fan D. Clinical application of electrocardiogram-guided tip positioning in peripheral inserted central catheters placement. *J Cancer Res Ther.* (2018) 14:887–91. 10.4103/jcrt.JCRT_46_18 29970671

[73] Elli S Bellani G Cannizzo L Giannini L De Felippis C Vimercati S Reliability of cutaneous landmarks for the catheter length assessment during peripherally inserted central catheter insertion: a retrospective observational study. *J Vasc Access.* (2020) 21:917–22. 10.1177/1129729820911225 32228229

[74] Mazzola JR Schott-Baer D Addy L. Clinical factors associated with the development of phlebitis after insertion of a peripherally inserted central catheter. *J Intraven Nurs.* (1999) 22:36–42. 10335176

[75] Nifong TP McDevitt TJ. The effect of catheter to vein ratio on blood flow rates in a simulated model of peripherally inserted central venous catheters. *Chest.* (2011) 140:48–53. 10.1378/chest.10-2637 21349931

